# Identification of the association between *FABP4* gene polymorphisms and milk production traits in Sfakia sheep

**DOI:** 10.5194/aab-62-413-2019

**Published:** 2019-07-15

**Authors:** Adel H. M. Ibrahim, Nikolaos Tzanidakis, Smaragda Sotiraki, Huitong Zhou, Jonathan G. H. Hickford

**Affiliations:** 1 Department of Animal Breeding, Desert Research Center, 1 Matehaf AlMatariya St., AlMatariya, Cairo 11753, Egypt; 2 Veterinary Research Institute, Hellenic Agriculture Organization, Thermi, TK 57001, Thessaloniki, Greece; 3 Gene-Marker Laboratory, Department of Agricultural Sciences, Lincoln University, POB 84, Lincoln 7647, New Zealand

## Abstract

The aim of this study was to estimate the effect of variation in the fatty acid binding protein 4 gene (*FABP4*) on milk production traits in Greek Sfakia sheep. Polymerase chain reaction – single-stranded conformational polymorphism (PCR-SSCP) analysis was used to genotype a total of 374 Sfakia ewes for two regions of *FABP4* located around exon 2–intron 2 (Region 1) and exon 3–intron 3 (Region 2). Each month, for a period of 6 months, milk samples were collected from the ewes to measure total milk yield, fat content, protein content, lactose content, non-fat solid content, pH, and somatic cell count (SCC). A general linear model was used to test the association between the variation observed in *FABP4* and milk production traits. Four gene variants (*A1*–*A4*) were found in Region 1 and two variants (*C1*–*C2*) were found in Region 2. In the first region, the *FABP4* genotype significantly affected (P<0.05) non-fat solid levels, fat content, and SCC. The presence of the *A2* variant was significantly associated (P<0.05) with decreased SCC, while the presence of *A4* was significantly associated with decreased milk yield (P<0.01), increased non-fat solid content (P<0.05), decreased fat content (P<0.01), increased lactose content (P<0.05), and increased pH (P<0.05). In the second region, *FABP4* genotype had an effect (P<0.05) on protein content and the presence of the *C2* variant was associated (P<0.05) with increased protein content, decreased SCC, and lower pH. The results suggest an association between variation in ovine *FABP4* and milk production traits in Greek Sfakia sheep. Nevertheless, further analyses in independent sheep populations of increased size will strengthen these findings.

## 1 Introduction

Sheep rearing for meat and milk production is an important part of the economy in the rural regions of Greece, contributing around 18 % to agricultural income and representing more than half of the country's animal production (Hadjigorgiou, 2014). The Sfakia breed is one of the most popular dual-purpose breeds of Greek sheep and its milk is used for consumption and for processing into cheese and yogurt. Sheep milk has advantages over cow milk because it contains higher levels of protein, lower levels of cholesterol, and large amounts of vitamins A and E, which act as antioxidants (Khan et al., 2019). With sheep milk, producing 1 kg of cheese takes approximately 4 kg of milk, compared to 8 to 10 kg for cows (Zeola et al., 2015).

Increasing the productivity of milking sheep is very important if the purpose is to increase the income of Greek farmers. Accordingly, it is important to understand the genetic basis of milk production traits. One approach, which has gained in popularity, is to search for the genetic basis of the important traits and select superior breeding stock using marker-assisted selection. The use of marker-assisted selection can speed up the identification of genetically superior animals by increasing the accuracy in which genetic merit can be ascertained, and by decreasing generation interval (Williams, 2005). To achieve this outcome, you first need to identify the major genes that affect any valued traits (Zhu and Zhao, 2007).

The various components of milk originate from blood plasma substrates and they are synthesized in the epithelial cells of the mammary gland (Kulig et al., 2013). Milk lipids are synthesized from fatty acids that bind to specific proteins called fatty acid binding proteins (FABPs). The FABPs are a small family of cytoplasmic proteins. They are thought to affect various cellular processes, in-particular lipid metabolism. They do this by transferring the fatty acids, heme, retinoids, and different vitamins across the cytoplasmic membrane to the sites of β-oxidation and triglyceride and phospholipid synthesis, and by modulating the concentration of the fatty acids in cells (Kulig et al., 2013).

To date, nine members of the *FABP* gene family (*FABP1*–*FABP9*) have been described. Fatty acid binding protein 4 (FABP4), also known as adipocyte FABP (A-FABP), is a protein found in abundance in the mammary gland, and also in mature adipocytes and adipose tissue (Hunt et al., 1986). The gene for FABP4 (*FABP4*) is expressed during lactation (Bionaz and Loor, 2008) and the main function of this protein is thought to be in lipid metabolism, where it binds both long-chain fatty acids and retinoic acid and delivers them to receptors in the nucleus of adipocytes (Spiegelman and Green, 1980).

Investigations of *FABP4*-deficient mice suggest that thermogenesis and whole-body energy expenditure are decreased after feeding on a high-fat diet, indicating the importance of *FABP4* in the maintenance of normal lipid metabolism (Cao et al., 2008). Furthermore, FABP4 was found to increase thermogenesis by promoting the conversion of thyroid hormone from its inactive form (T4) to active form (T3) in brown adipocytes, the levels of *FABP4* were found to increase in the bloodstream, and in both brown and white adipose tissues, in response to thermogenic stimuli (Shu et al., 2017). In this respect, it has been shown that the performance of lactating animals is not only limited by the intrinsic properties of mammary glands but also by competition from heat production including thermogenesis in brown adipose tissue (Król et al., 2011). Taken together, these suggest that the performance of lactating ewes could be affected by *FABP4*.

The FABP4 gene has been identified in sheep, cattle, chickens, and humans. Across these species it has a conserved structure with four exons being separated by three introns. Studies on whether variation in *FABP4* affects milk production traits in sheep have not been undertaken but Yan et al. (2012) analyzed two regions of the gene (Region 1: exon 2–intron 2; and Region 2: exon 3–intron 3) using polymerase chain reaction – single-stranded conformational polymorphism (PCR-SSCP) and DNA sequencing. They detected five different SSCP patterns derived from three nucleotide substitutions and one deletion in Region 1 and four different SSCP patterns derived from four nucleotide substitutions in Region 2.

It is assumed that the lipid metabolism, thermogenesis, and whole-body energy expenditure affects most biological functions in the cells of various tissues and organs, including the mammary gland; therefore, *FABP4* is considered to be a candidate gene for milk production traits in sheep. The objective of the present study was therefore to look for genetic variation in two separate regions of ovine *FABP4* gene and, if found, test its association with milk production traits in Greek Sfakia ewes.

## 2 Materials and methods

### 2.1 Animal sources and experimental design

Twenty (n=20) different flocks of the Sfakia breed, from the provinces of Rethymno and south Chania on Crete, were investigated. Ten flocks were representative of a semi-intensive production system and the other 10 were representative of the more traditional extensive production system. Within each flock, 18–20 ewes (in their second or third lactation) were randomly chosen for analysis. Eight to 10 ewes were randomly chosen from the early lambing period in autumn, when the multiparous ewes lamb, and 9 to 10 ewes were randomly chosen from the late lambing period in winter, when the primiparous ewes lamb.

### 2.2 Phenotypic measurements and analytical methods

All ewes were milked twice daily and the daily milk yield was measured using graduated measuring cylinders. Milk samples for analysis were collected once a month for a period of 6 months. These samples were analyzed at the State Milk Quality Laboratory (ELOGAK) in Rethymno. First, the samples were heated to 25 ${}^{\circ }$C and the pH measured. For samples with a pH above 6.0, fat percentage, protein percentage, lactose percentage, and non-fat solid percentage were assessed by infrared analysis using a MilkoScan^™^ (FT, FOSS^®^, Hillerød, Denmark) and by flow cell cytometry for somatic cell count (SCC) using the Fossomatic^™^ (FC, FOSS^®^). Fat, protein, lactose, and non-fat solid contents in the milk were expressed in grams per 100 mL and the logarithmic value of somatic cell count in milk was recorded.

**Table 1:** List of primer sequences used for PCR.

Region amplified	Size (bp)	Primer sequence	Reference
Region 1	350	F: CAGGAATTTGATGAAGTCACT	Yan et al. (2012)
(exon 2–intron 2)		R: GTAACATGGTTCAGAGCTAG	
Region 2	524	F: GATGGGAAATCAACCACCA	Yan et al. (2012)
(exon 3–intron 3)		R: TCTCCTTCAATGCTGAGAAG	

F = forward; R = reverse.

### 2.3 PCR-SSCP analysis and genotyping of *FABP4*

Blood samples were collected from 374 Sfakia ewes onto FTA cards (Flinders Technology Associates); genomic DNA was purified for PCR analysis using a two-step procedure that is described by Zhou et al. (2006). Two pairs of specific primers (Table 1) were used to study the genetic variation in two regions of the *FABP4* gene (Region 1 and Region 2) that are located on exon 2–intron 2 and exon 3–intron 3.

The PCR reaction was carried out in a total reaction volume of 20 µL containing the DNA on a 1.2 mm punch of FTA card, 0.25 µM of each primer, 2.5 µL of 10× PCR buffer, 1.5 mM of ${\mathrm{MgCl}}_{2}$, 150 µM of each dNTP (Eppendorf, Hamburg, Germany), and 0.5 U (one unit) of *Taq* DNA polymerase (Qiagen, Hilden, Germany). The thermal profile consisted of a 2 min denaturation at 94 ${}^{\circ }$C followed by 35 cycles of 30 s at 94 ${}^{\circ }$C, 30 s at 60 ${}^{\circ }$C, and 30 s at 72 ${}^{\circ }$C, and with a final extension step of 5 min at 72 ${}^{\circ }$C. The PCR products were subject to single stranded conformational polymorphism (SSCP) analysis in 14 % polyacrylamide gels at 320 V and 12 ${}^{\circ }$C for 18 h in 0.5× TBE buffer, and the gels were silver-stained using the method of Byun et al. (2009).

### 2.4 Sequencing and analysis of the sequence variation

The amplicons from two sheep that produced each homozygous SSCP pattern for *FABP4* were used as templates for Sanger sequencing of DNA at the Lincoln University DNA Sequencing Facility. For each heterozygous SSCP pattern, two sheep were sequenced using the method of Gong et al. (2011). Briefly, one of the unique bands of the heterozygous pattern was cut out of the gel, washed with 300 µL 1× TE buffer, mashed up with a micropipette tip in 30 µL 1× TE buffer and incubated for 50 min at 55 ${}^{\circ }$C. This product was used as the DNA template for re-amplification using the PCR conditions described above. The re-amplified DNA was purified using a PCR purification kit (Thermo Fisher Scientific, Waltham, MA, USA) and then sequenced in both directions. DNA sequence analyses, including translation to amino acid sequence and sequence comparisons, were undertaken using DNAMAN software (version 7.212, Lynnon Corp., QC, Canada) and DNASTAR software (Madison, WI, USA).

### 2.5 Statistical analyses

Data were analyzed using the general linear model (GLM) procedure of SAS software (SAS, 2004) to estimate the effect of *FABP4* genotype, lactation number, lambing period, and management system. Sheep with *FABP4* genotypes with frequencies less than 4 % were omitted from the statistical analysis to reduce the chances of obtaining false positive results during the multiple comparisons.

The mathematical model used can be written as follows:

$$Y_{ijklm}=\mu +G_{i}+B_{j}+C_{k}+{\mathrm{FM}}_{l}+e_{ijklm},$$

where $Y_{ijklm}$ is the observed trait value in the ijklmth animal, μ is the mean trait value for a given trait, $G_{i}$ is the fixed effect of the ith *FAPB4* genotype (i=1 to 5 for the first region and i=1 to 3 for the second region) or the fixed effect of the presence or absence of each detected *FAPB4* variant (i=0, 1 for each variant, in each region), $B_{j}$ is the effect of lactation number (j=2, 3), $C_{k}$ is effect of lambing period (k=1,2), FM${}_{l}$ is the fixed effect of lth farm (1,…10) for each management system (1, 2), and $e_{ijklm}$ is the random error.

The differences between mean trait values were verified by the Duncan test (P≤0.05).

**Figure 1:**
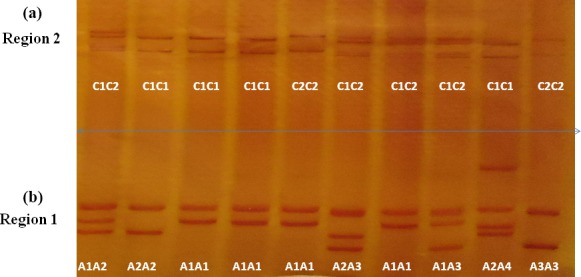
PCR-SSCP for exon 2–intron 2 **(b)** and exon 3–intron 3 **(a)** of the *FABP4* gene in Sfakia ewes.

**Table 2:** Sequence variation in the two regions of ovine *FABP4*.

	Position	Nucleotide sequence			
		*A1*	*A2*	*A3*	*A4*
Region 1	c.246+34	C	–	C	C
	c.246+37	G	A	A	A
	c.246+46	C	C	C	T
	c.246+47	G	G	G	G
	Position	Nucleotide sequence			
		*C1*		*C2*	
Region 2	c.348+298	T		C	
	c.348+356	T		C	

## 3 Results

### 3.1 Identification of sequence variation in *FABP4*

**Figure 2:**
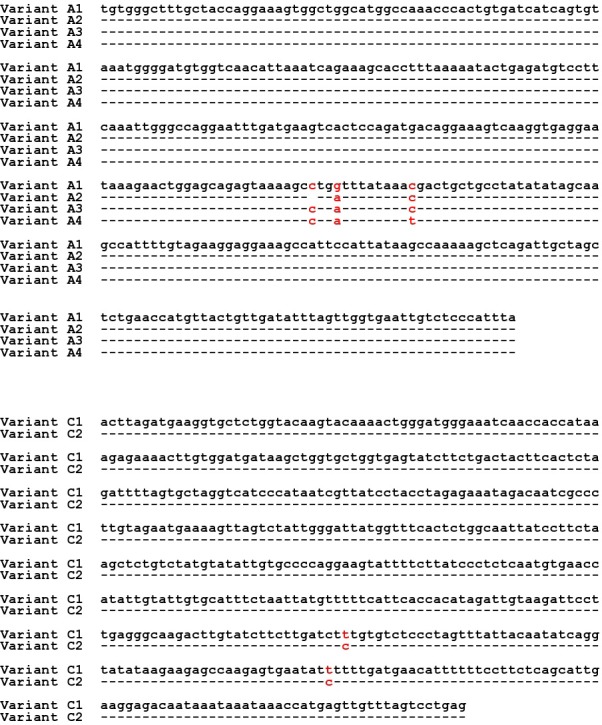
Sequences of the detected variants in Region 1 (*A1, A2, A3*, and *A4*) and Region 2 (*C1* and *C2*) of the FABP4 gene in Sfakia sheep.

Four different SSCP banding patterns were observed for amplicons derived from the exon 2–intron 2 region of *FABP4* in the Sfakia sheep, and 10 combinations of these banding patterns corresponded to 10 different genotypes (named: *A1A1*, *A1A2*, *A1A3*, *A1A4*, *A2A2*, *A2A3*, *A2A4*, *A3A3*, *A3A4*, and *A4A4*; Fig. 1). These had frequencies of 17.1 %, 31.6 %, 14.4 %, 6.3 %, 15.9 %, 8.8 %, 3.8 %, 0.7 %, 0.7 %, and 0.5 %, respectively. They were comprised of four unique variant sequences (*A1*, *A2*, *A3*, and *A4*; Table 2 and Fig. 2) with frequencies of 43.3 %, 38.0 %, 12.8 %, and 5.9 %, respectively.

The variant frequencies for the second region of *FABP4* in the Sfakia sheep were 73.0 % and 27.0 % for variants that were named *C1* and *C2*, respectively. Three genotypes were observed: *C1C1* (frequency 53.0 %), *C1C2* (40.0 %), and *C2C2* (7.0 %) (Table 2 and Fig. 2).

### 3.2 Effect of nongenetic factors on milk production traits

The nongenetic factors (lactation number, lambing period, and farm-management system) significantly (P<0.05, P<0.01) affected all of the milk production traits for the Sfakia ewes.

**Table 3:** Least square means and their standard error (SE) for milk production traits in Greek Sfakia ewes according to the *FABP4* genotypes.

Genotype	n	Milk yield		Non-fat solids		Protein		Fat		Lactose		pH		SCC	
				(mg per 100 mL)		(mg per 100 mL)		(mg per 100 mL)		(mg per 100 mL)				(log)	
		Estimate	SE	Estimate	SE	Estimate	SE	Estimate	SE	Estimate	SE	Estimate	SE	Estimate	SE
Region 1															
*A1A1*	68	561	14	10.81${}^{\text{ab}}$	0.08	5.36	0.06	5.07${}^{\text{b}}$	0.11	4.59	0.06	6.79	0.02	5.29${}^{\text{ab}}$	0.06
*A1A2*	125	587	10	10.77${}^{\text{b}}$	0.06	5.29	0.04	5.21${}^{\text{ab}}$	0.08	4.66	0.04	6.78	0.01	5.16${}^{\text{b}}$	0.04
*A1A3*	57	576	16	10.74${}^{\text{b}}$	0.09	5.32	0.07	5.03${}^{\text{c}}$	0.12	4.60	0.07	6.81	0.02	5.36${}^{\text{a}}$	0.07
*A1A4*	25	553	24	10.98${}^{\text{a}}$	0.19	5.29	0.12	5.01${}^{\text{c}}$	0.17	4.58	0.09	6.82	0.03	5.15${}^{\text{b}}$	0.10
*A2A2*	63	596	16	10.91${}^{\text{ab}}$	0.09	5.37	0.06	5.22${}^{\text{ab}}$	0.11	4.71	0.06	6.79	0.02	5.16${}^{\text{b}}$	0.06
*A2A3*	35	609	20	10.65${}^{\text{c}}$	0.16	5.19	0.08	5.32${}^{\text{a}}$	0.15	4.64	0.08	6.77	0.02	5.27${}^{\text{ab}}$	0.08
P value		*0.082*		0.015		0.369		0.047		0.549		0.923		0.021	
Region 2															
*C1C1*	210	584	8	10.84	0.05	5.25${}^{\text{c}}$	0.03	5.17	0.06	4.65	0.03	6.78	0.01	5.15	0.03
*C1C2*	159	577	9	10.70	0.05	5.36${}^{\text{b}}$	0.04	5.11	0.07	4.62	0.04	6.80	0.01	5.28	0.04
*C2C2*	25	598	23	10.78	0.14	5.41${}^{\text{a}}$	0.10	5.28	0.17	4.53	0.10	6.80	0.03	5.30	0.10
P value		0.262		0.196		0.039		0.263		0.994		0.355		*0.051*	

SCC: somatic cell count. SE: standard error. Means within a column that do not share a superscript letter are significantly (P<0.05) different. Means followed by different letter(s) within a column are significantly different (P<0.05) according to Duncan's adjustment for pairwise comparisons. Means not followed by letter(s) within a column are not significantly different (P<0.05) according to Duncan's adjustment for pairwise comparisons.

### 3.3 Effect of *FABP4* genotype in the first region on milk traits

Associations between the *FABP4* genotypes in the first region and the milk production traits are summarized in Table 3. Associations (P<0.05) were observed between *FABP4* genotypes in the first region and non-fat milk solids, fat content, and SCC. Ewes with the *A1A4* genotype had higher non-fat solid levels, lower fat content, and lower SCC, while ewes with the *A2A3* genotype had lower non-fat solid levels and higher fat content. Furthermore, a trend (P=0.082) was observed for an association between the *FABP4* genotype in the first region and milk yield. Ewes with the *A1A4* genotype had lower milk yields, while the *A2A3* ewes had higher milk yields.

**Table 4:** Least square means and their standard errors for milk production traits in Greek Sfakia ewes according to the presence/absence of FABP4 variants.

Variant		n	Milk yield		Non-fat solids		Protein		Fat		Lactose		pH		SCC	
					(mg per 100 mL)		(mg per 100 mL)		(mg per 100 mL)		(mg per 100 mL)				(log)	
			Estimate	SE	Estimate	SE	Estimate	SE	Estimate	SE	Estimate	SE	Estimate	SE	Estimate	SE
Region 1																
*A1*	Absent	114	594	10	10.60	0.06	5.34	0.04	5.20	0.08	4.63	0.04	6.79	0.01	5.20	0.04
	Present	259	577	7	10.58	0.03	5.31	0.03	5.13	0.05	4.64	0.03	6.79	0.01	5.22	0.03
*A2*	Absent	148	569	9	10.62	0.05	5.33	0.04	5.07	0.07	4.63	0.04	6.79	0.01	5.24	0.04
	Present	225	591	7	10.56	0.04	5.31	0.03	5.21	0.05	4.64	0.03	6.79	0.01	5.19*	0.03
*A3*	Absent	282	580	7	10.58	0.03	5.33	0.03	5.16	0.05	4.63	0.03	6.79	0.01	5.19	0.03
	Present	91	589	12	10.60	0.06	5.28	0.05	5.12	0.09	4.63	0.05	6.78	0.01	5.27	0.05
*A4*	Absent	330	584	6	10.57	0.03	5.31	0.03	5.17	0.04	4.56	0.03	6.79	0.01	5.22	0.03
	Present	43	570**	18	10.72*	0.10	5.37	0.08	5.00**	0.13	4.64*	0.08	6.80*	0.02	5.14	0.08
Region 2																
*C1*	Absent	23	599	23	10.79	0.14	5.42	0.10	5.29	0.17	4.53	0.10	6.67	0.02	5.29	0.10
	Present	350	581	6	10.78	0.03	5.31	0.02	5.14	0.04	4.64	0.02	6.68	0.01	5.21	0.03
*C2*	Absent	196	586	8	10.84	0.05	5.25	0.03	5.13	0.06	4.61	0.04	6.69	0.01	5.27	0.04
	Present	177	578	9	10.72	0.05	5.36*	0.04	5.17	0.06	4.65	0.03	6.67*	0.01	5.14	0.03*

${}^{*}$ refers to significance at (P<0.05); ${}^{**}$ refers to significance at (P<0.01); SCC: somatic cell count; SE: standard error.

The results of testing the association between absence or presence of the *FABP4* variants of the first region in ewe genotype and milk production traits are presented in Table 4. These results reveal that the presence of *A2* in the genotyped ewes was associated with decreased SCC (P<0.05), while the presence of *A4* in the ewes was associated with decreased milk yield (P<0.01), increased non-fat solid content (P<0.05), decreased fat content (P<0.01), increased lactose content (P<0.05), and increased pH (P<0.05).

### 3.4 Effect of FABP4 genotype in the second region on milk traits

The results in Table 3 reveal that *FABP4* genotype in the second region had an effect on protein content (P<0.05). A comparison of the milk production traits for the *FABP4* genotypes proved that the ewes with the *C2C2* genotype had the highest protein content, while the ewes with the *C1C1* genotype had the lowest protein content. As shown in Table 4, the presence of the *C2* variant in the ewe genotype was associated (P<0.05) with an increase in protein content, and decreases in pH, and SCC.

## 4 Discussion

This is the first report describing the effect of *FABP4* variation on milk production traits in sheep. In the study, a PCR-SSCP method proved to be a robust and reliable way to identify genetic markers associated with milk traits in Sfakia sheep. It unambiguously revealed 10 combinations of four PCR-SSCP patterns that were made up of four unique DNA sequences from the exon 2–intron 2 region of *FABP4* and three combinations of two PCR-SSCP patterns representing two unique DNA sequences from a part of exon 3 and intron 3. On the basis of sequence comparison, the FABP4 variants *A1*, *A2*, *A3*, and *A4* described here appeared to match variants *A1*, *B1*, *C1,* and *D1*, respectively, as described by Yan et al. (2012) and listed in GenBank with accession numbers JX290313-JX290316. It is evident from Tables 3 and 4 that there are significant associations for the variation observed in Region 1 of *FAPB4* with milk yield, non-fat milk solid content, fat content, lactose content, pH, and SCC. While the nucleotide substitutions that were found in this region are located in intron 2, and do not change the amino acid sequence, it is possible that these substitutions might still influence *FABP4* expression. For example they might affect the splicing of mRNA or be linked to variation elsewhere in a regulatory region (e.g., a miRNA binding site or enhancer binding site), or the 5${}^{\prime }$–UTR or 3${}^{\prime }$–UTR regions (UTR is untranslated region), that subsequently affects expression of the amino acid sequence (Wessagowit et al., 2005; MartõÂnez-Pizarro et al., 2018).

Previous reports have demonstrated associations between genetic variation in *FABP4* and carcass traits, meat quality traits, and fatty acid composition in adipocytes (Ardicli et al., 2017; Yan et al., 2018). Limited information is available regarding the effect of *FABP4* on milk production traits in livestock. In Jersey cattle, Kulig et al. (2010) studied variation in two regions of the bovine *FABP4* gene (from nucleotides 5433 to 6106 and from nucleotides 7417 to 7868), for the accession number AAFC01136716, and found no associations between the *FABP4 *polymorphism and daily milk yield, percentage fat content, or percentage protein content. For the same regions of *FABP4* in Polish Holstein-Friesian cows, Kulig et al. (2013) revealed a significant effect for the single-nucleotide polymorphism (SNP; c.328G > A) on the estimated breeding values for protein yield (P<0.05) and percentage protein content (P<0.01). The same SNP (c.328G > A) was tested in Polish Holstein-Friesian black-white cows by Kaczor et al. (2017), but they did not find any effects for this SNP on milk performance traits. Nafikov et al. (2013) sequenced the exonic and some intronic regions of *FABP4* to discover the SNPs and haplotypes associated with milk fat percentage and fatty acid composition and reported that the overall haplotype effect of *FABP4* was significantly associated with the concentrations of saturated fatty acids (SFA), un-saturated fatty acids (UFA), monounsaturated fatty acids (MUFA), polyunsaturated fatty acids (PUFA), and the SFA/UFA ratio. Zhou et al. (2015) identified three PCR-SSCP patterns derived from five SNPs: two of these SNPs were detected in exon 3 and the other SNPs were detected in intron 3; these five SNPs defined three haplotype sequences (named A, B, and C) and these were associated with milk yield and protein percentage in Holstein-Friesian × Jersey-cross dairy cows.

Comparison of the exon 3–intron 3 sequences in this study with the sequences described by Yan et al. (2012) revealed that the *FABP4* variants *C1* and *C2* from the Sfakia sheep have the same sequences as two variants previously described in many New Zealand breeds of sheep (named *A2* and *D2*, GenBank accession numbers JX409931 and JX409934, respectively; Table 2 and Fig. 2). It is notable that the *C2* variant, which is associated with increased protein content, decreased SCC and lower pH, and carries the nucleotide substitution c.317A/G that would lead to the putative amino acid substitution of lysine with arginine at residue 106. Arginine is one of the most multifunctional amino acids in animal cells. It turns into polyamines, which play a key role in the synthesis of protein in mammary epithelial cells (Kim and Wu, 2009). Previous studies revealed the importance of arginine in increasing milk protein content. For example, Moreira et al. (2018) described how dietary supplementation with high levels of arginine increased milk production and milk protein in pigs. Clark et al. (1975) found that arginine uptake by the mammary gland associated with milk yield in rabbits and cows; however, O'Quinn et al. (2002) revealed that the rate of arginine catabolism in the lactating sows was greater than that for the pregnant or non-lactating sows. Furthermore, Tian et al. (2017) stated that arginine deficiencies had negative effects on milk yield and milk protein yield. The investigators attributed the effect of arginine to its crucial role in regulating protein turnover in mammary epithelial cells by activating the mechanistic target of the rapamycin (mTOR) cell signaling pathway (Ma et al., 2018).

The lower SCC for milk produced from ewes which carry the *C2* variant might be attributable to the substitution of lysine with arginine in FABP4. Many studies have described a positive effect for arginine on reducing the SCC in milk produced from mammals. For example, Chacher et al. (2013) suggested that promotion of the synthesis and metabolism of endogenous L-Arginine, through supplementation with feed additives that elevate blood plasma arginine levels, enhanced immunity, reduced SCC, and reduced the incidence of mastitis in sows. McCoard et al. (2013) found that administration of L-arginine to Romney ewes improved mammary gland health and decreased SCC in the milk produced. Woloszyn (2007) revealed that the cows with high SCC had a significantly lower concentration of arginine in their blood plasma. Alternatively, studies (Troendle et al., 2016; Hachana et al., 2018) have described a positive correlation between the number of somatic cells in milk and its pH. Given that the ewes that carry the *C2* variant also produced milk with lower pH, the effect of *C2* on SCC may either be mediated via pH or arginine levels.

The effects of *FABP4 *variation on milk production traits might also be explained by other phenomena. Besides its direct effects on milk production traits, *FABP4* has been shown to have an effect on residual feed intake and feed efficiency (Cohen-Zinder et al., 2016), subcutaneous fat depth and fatty acid composition of carcass (Hoashi et al., 2008), intramuscular fat (Lee et al., 2010), marbling score and back-fat thickness (Ardicli et al., 2017), and insulin resistance (Hotamisligil and Bernlohr, 2015). Many investigations have shown associations between milk production traits and these traits. Casper (2008) stated that the lower residual feed intake was, the higher feed efficiency was, leading to increased milk production in dairy cows. Marston et al. (1998) speculated that increased milk production was positively associated with greater marbling in beef cows. A strong relationship was found between the development of back-fat thickness and milk protein content in dairy cows (Schröder and Staufenbiel, 2003). A positive correlation of milk yield and milk fat content, with increased subcutaneous fat depth, in dairy cows was revealed by Nogalski et al. (2012). Elevated levels of insulin in plasma were also found to be associated with decreases in milk production and milk lactose content and increases in milk fat and protein contents in Holstein dairy cows and goats (Mackle et al., 1999; Bequette et al., 2001).

## 5 Conclusions

It could be concluded that the variation in ovine *FABP4* has an effect on milk production traits. However, further investigations have to be undertaken on a larger population of Sfakia sheep, or other breeds of sheep, to confirm these findings before they can be recommended to breeding programs to improve milk production traits in sheep.

## Data Availability

In 2013 Nikolaos Tzanidakis visited the Gene-marker Laboratory, Lincoln University, New Zealand, to carry out extensive research to identify genetic markers associated with milk production traits in Sfakia sheep. Unfortunately, the knee of Nikolaos Tzanidakis was broken in an accident and he was obligated to come back to Greece. Then, Jonathan G. H. Hickford, in collaboration with Nikolaos Tzanidakis, asked me (Adel H. M. Ibrahim, from Egypt) to continue this work. Therefore, I (Adel H. M. Ibrahim) have no right to share the Greek sheep data.

## References

[bib1.bib1] Ardicli S, Samli H, Alpay F, Dincel D, Soyudal B, Balci F (2017). Association of single nucleotide polymorphisms in the FABP4 gene with carcass characteristics and meat quality in Holstein bulls. Ann Anim Sci.

[bib1.bib2] Bequette BJ, Kyle CE, Crompton LA, Buchan V, Hanigan MD (2001). Insulin regulates milk production and mammary gland and hind-leg amino acid fluxes and blood flow in lactating goats. J Dairy Sci.

[bib1.bib3] Bionaz M, Loor JJ (2008). ACSL1, AGPAT6, FABP3, LPIN1, and SLC27A6 are the most abundant isoforms in bovine mammary tissue and their expression is affected by stage of lactation. J Nutr.

[bib1.bib4] Byun SO, Fang Q, Zhou H, Hickford JG (2009). An effective method for silver-staining DNA in large numbers of polyacrylamide gels. Anal Biochem.

[bib1.bib5] Cao H, Gerhold K, Mayers JR, Wiest MM, Watkins SM, Hotamisligil GS (2008). Identification of a lipokine, a lipid hormone linking adipose tissue to systemic metabolism. Cell.

[bib1.bib6] Casper DP (2008). Factors Affecting Feed Efficiency of Dairy Cows.

[bib1.bib7] Chacher B, Liu H, Wang D, Liu J (2013). Potential role of N-carbamoyl glutamate in biosynthesis of arginine and its significance in production of ruminant animals. J Anim Sci Biotechnol.

[bib1.bib8] Clark JH, Derrig RG, Davis CL, Spires HR (1975). Metabolism of arginine and ornithine in the cow and rabbit mammary tissue. J Dairy Sci.

[bib1.bib9] Cohen-Zinder M, Asher A, Lipkin E, Feingersch R, Agmon R, Karasik D, Brosch A, Shabtay A (2016). FABP4 is a leading candidate gene associated with residual feed intake in growing Holstein calves. Physiol Genomics.

[bib1.bib10] Gong H, Zhou H, Hickford JG (2011). Diversity of the glycine/tyrosine-rich keratin-associated protein 6 gene (KAP6) family in sheep. Mol Biol Rep.

[bib1.bib11] Hachana Y, Znaidi A, M'Hamadi N (2018). Effect of somatic cell count on milk composition and mozzarella cheese quality. Acta Aliment.

[bib1.bib12] Hadjigorgiou I (2014). Sheep and goat farming and rural development in Greece.

[bib1.bib13] Hoashi S, Hinenoya T, Tanaka A, Ohsaki H, Sasazaki S, Taniguchi M, Oyama K, Mukai F, Mannen H (2008). Association between fatty acid compositions and genotypes of FABP4 and LXR-alpha in Japanese Black cattle. BMC Genet.

[bib1.bib14] Hotamisligil GS, Bernlohr DA (2015). Metabolic functions of FABPs – mechanisms and therapeutic implications. Nat Rev Endocrinol.

[bib1.bib15] Hut CR, Ro JH, Dobson DE, Min HY, Spiegelman BM (1986). Adipocyte P2 gene: developmental expression and homology of 5
${}^{\prime }$
-flanking sequences among fat cell-specific genes. P Natl Acad Sci USA.

[bib1.bib16] Kaczor U, Famielec M, Dudziak P, Kaczor A, Kucharski M, Mandecki A (2017). Fatty acid binding protein 4 (FABP4) and thyroglobulin (TG)
polymorphisms in relation to milk performance traits in the
Holstein-Friesian cattle. Acta Sci Pol Zootech.

[bib1.bib17] Khan IT, Nadeem M, Imran M, Ullah R, Ajmal M, Jaspal MH (2019). Antioxidant properties of milk and dairy products: A comprehensive review of the current knowledge. Lipids Health Dis.

[bib1.bib18] Kim SW, Wu G (2009). Regulatory role for amino acids in mammary gland growth and milk synthesis. Amino Acids.

[bib1.bib19] Król E, Martin SA, Huhtaniemi IT, Douglas A, Speakman JR (2011). Negative correlation between milk production and brown adipose tissue gene expression in lactating mice. J Exp Biol.

[bib1.bib20] Kulig H, Kowalewska-Łuczak I, Kmieć M, Wojdak-Maksymiec K (2010). ANXA9, SLC27A3, FABP3 and FABP4 single nucleotide polymorphisms in relation to milk production traits in Jersey cows. Czech J Anim Sci.

[bib1.bib21] Kulig H, Kowalewska-Luczak I, Zukowski K, Kruszyński W (2013). FABP3, FABP4 and ANXA9 SNP genotypes in relation to breeding values for milk production traits in Polish Holstein-Friesian cows. Genetika.

[bib1.bib22] Lee SH, van der Werf JH, Kim NK, Lee SH, Gondro C, Park EW, Oh SJ, Gibson JP, Thompson JM (2011). QTL and gene expression analyses identify genes affecting carcass weight and marbling on BTA14 in Hanwoo (Korean Cattle). Mamm Genome.

[bib1.bib23] Ma Q, Hu S, Bannai M, Wu G (2018). L-Arginine regulates protein turnover in porcine mammary epithelial cells to enhance milk protein synthesis. Amino Acids.

[bib1.bib24] Mackle TR, Dwyer DA, Ingvartsen KL, Chouinard PY, Lynch JM, Barbano DM, Bauman DE (1999). Effects of insulin and amino acids on milk protein concentration and yield from dairy cows. J Dairy Sci.

[bib1.bib25] Marston T, Gleghorn JF, Wankel LE (1998). The impact of selecting for marbling on beef cows herds, Agricultural Experiment Station and Cooperative Extension Service, April.

[bib1.bib26] MartõÂnez-Pizarro A, Dembic M, PeÂrez B, Andresen BS, Desviat LR (2018). Intronic *PAH* gene mutations cause a splicing defect by a novel mechanism involving U1snRNP binding downstream of the 5' splice site. PLoS Genet.

[bib1.bib27] McCoard S, Sales F, Wards N, Sciascia Q, Oliver M, Koolaard J, van der Linden D (2013). Parenteral administration of twin-bearing ewes with L-arginine enhances the birth weight and brown fat stores in sheep. SpringerPlus.

[bib1.bib28] Moreira RH, Lanferdini E, Fonseca LS, Chaves RF, Garbossa CA, Saraiva A, Nogueira ET, de Abreu ML (2018). Arginine improves nutritional quality of sow milk and piglet performance. Braz J Ani Sci.

[bib1.bib29] Nafikov RA, Schoonmaker JP, Korn KT, Noack K, Garrick DJ, Koehler KJ, Minick-Bormann J, Reecy JM, Spurlock DE, Beitz DC (2013). Association of polymorphisms in solute carrier family 27, isoform A6 (SLC27A6) and fatty acid-binding protein-3 and fatty acid-binding protein-4 (FABP3 and FABP4) with fatty acid composition of bovine milk. J Dairy Sci.

[bib1.bib30] Nogalski Z, Wroński M, Sobczuk-Szul M, Mochol M, Pogorzelska P (2012). The effect of body energy reserve mobilization on the fatty acid profile of milk in high-yielding cows. Asian-Austral J Anim Sci.

[bib1.bib31] O'Quinn PR, Knabe DA, Wu G (2002). Arginine catabolism in lactating porcine mammary tissue. J Anim Sci.

[bib1.bib32] Schröder U, Staufenbiel R (2003). Relationships between backfat thickness, milk yield and fertility traits with resulting standard curves and their application in dairy herd management. Acta Vet Scand.

[bib1.bib33] Shu L, Hoo RL, Wu X, Pan Y, Lee IP, Cheong LY, Bornstein SR, Rong X, Guo J, Xu A (2017). A-FABP mediates adaptive thermogenesis by promoting intracellular activation of thyroid hormones in brown adipocytes. Nat Commun.

[bib1.bib34] Spiegelman BM, Green H (1980). Control of specific protein biosynthesis during the adipose conversion of 3T3 cells. J Biol Chem.

[bib1.bib35] Tian W, Wu T, Zhao R, Xu J, He Y, Wang H (2017). Responses of milk production of dairy cows to jugular infusions of a mixture of essential amino acids with or without exclusion leucine or arginine. Anim Nutr.

[bib1.bib36] Troendle JA, Tauer LW, Grohn TG (2016). Optimally achieving milk bulk tank somatic cell count thresholds. J Dairy Sci.

[bib1.bib37] Wessagowit V, Nalla VK, Rogan PK, McGrath JA (2005). Normal and abnormal mechanisms of gene splicing and relevance to inherited skin diseases. J Dermatol Sci.

[bib1.bib38] Williams JL (2005). The use of marker-assisted selection in animal breeding and biotechnology. Revue Sci Tech.

[bib1.bib39] Woloszyn M (2007). Natural Variations of Milk Somatic Cell Count in Dairy Cows[pHD].

[bib1.bib40] Yan W, Zhou H, Luo Y, Hu J, Hickford JG (2012). Allelic variation in ovine fatty acid-binding protein (FABP4) gene. Mol Biol Rep.

[bib1.bib41] Yan W, Zhou H, Hu J, Luo Y, Hickford JG (2018). Variation in the FABP4 gene affects carcass and growth traits in sheep. Meat Sci.

[bib1.bib42] Zeola NM, Sobrinho AG, Hatsumura CT, Borghi TH, Viegas CR, Barbosa JC (2015). Production, composition and processing of milk from ewes fed soybean Seeds. R Bras Zootec.

[bib1.bib43] Zhou H, Cheng L, Azimu W, Hodge S, Edwards GR, Hickford JG (2015). Variation in the bovine FABP4 gene affects milk yield and milk protein content in dairy cows. Sci Rep.

[bib1.bib44] Zhou H, Hickford JG, Fang Q (2006). A two-step procedure for extracting genomic DNA from dried blood spots on filter paper for polymerase chain reaction amplification. Anal Biochem.

[bib1.bib45] Zhu M, Zhao S (2007). Candidate Gene Identification Approach: Progress and Challenges. Int J Biol Sci.

